# Soil warming enhances the hidden shift of elemental stoichiometry by elevated CO_2_ in wheat

**DOI:** 10.1038/srep23313

**Published:** 2016-03-22

**Authors:** Xiangnan Li, Dong Jiang, Fulai Liu

**Affiliations:** 1 University of Copenhagen, Faculty of Science, Department of Plant and Environmental Sciences, Højbakkegaard Allé 13, 2630 Tåstrup, Denmark; 2 National Engineering and Technology Center for Information Agriculture/Key Laboratory of Crop Physiology and Ecology in Southern China, Ministry of Agriculture, Nanjing Agricultural University, Nanjing, China

## Abstract

Increase in atmospheric CO_2_ concentration ([CO_2_]) and associated soil warming along with global climate change are expected to have large impacts on grain mineral nutrition in wheat. The effects of CO_2_ elevation (700 μmol l^−1^) and soil warming (+2.4 °C) on K, Ca and Mg concentrations in the xylem sap and their partitioning in different organs of wheat plant during grain filling were investigated. Results showed that the combination of elevated [CO_2_] and soil warming improved wheat grain yield, but decreased plant K, Ca and Mg accumulation and their concentrations in the leaves, stems, roots and grains. The reduced grain mineral concentration was attributed to the lowered mineral uptake as exemplified by both the decreased stomatal conductance and mineral concentration in the xylem sap. These findings suggest that future higher atmospheric [CO_2_] and warmer soil conditions may decrease the dietary availability of minerals from wheat crops. Breeding wheat cultivars possessing higher ability of mineral uptake at reduced xylem flux in exposure to climate change should be a target.

Wheat (*Triticum aestivum* L.) is one of the most important agricultural food crops worldwide, providing around 20% of protein in the human diet. It is also an important source for minerals^1^. However, the human nutrition depending on wheat grains might be threatened by the climate change^2^. The increasing atmospheric CO_2_ concentration (hereafter abbreviated to [CO_2_]) has been reported to decrease the concentrations of minerals, including the macro-elements, such as magnesium (Mg), calcium (Ca) and potassium (K), micro-elements and trace elements in wheat grains^3^,^4^.

Several reasons may account for the reduced mineral nutrition of wheat grains under CO_2_ elevation. Dilution by increased accumulation of carbohydrates under high [CO_2_] is a likely cause, but it cannot explain all micronutrient reductions well^5^. For instance, grain mass was not influenced by CO_2_ elevation, whereas cadmium concentration was lowered^4^. On the other hand, limited uptake of nutrients by roots under elevated [CO_2_] could be another possible reason^6^,^7^. This reduced root uptake could be related to factors influencing plant transpiration, since most of minerals are transported from root to shoot in the transpiration stream via the xylem^5^. The nutrient transport rate in the xylem is mainly determined by two independent factors: the sap flow rate and the concentration of nutrients in the xylem sap^6^,^8^. In exposure to elevated [CO_2_], wheat increases photosynthesis but reduces the stomatal conductance resulting in lower transpiration rate^9^,^10^. The lowered transpiration rate could limit the transport of minerals from the root to shoot, hereby reduce their concentration in the grains^5^. However, the effect of elevated [CO_2_] on xylem concentrations of minerals and their relations to nutrient loading to the grains in wheat remain largely elusive.

The possibility of changed mineral uptake by root should also be directly related to the changes in whole-plant growth including roots and the allocation of minerals to the different organs under elevated [CO_2_]^11^,^12^. In particular, elevated [CO_2_] and the associated global warming have been reported to have significant effects on physiological processes and grain yield in wheat^13^,^14^. The temperature trend under climate change is indicated as not only air temperature increase but also rise in soil temperature, which has been reported to limit wheat crop development and yield^15^,^16^, especially the root growth^17^. In barley, the concentrations of K and Ca in grain were increased with soil warming (+2.5 °C), while concentration of Mg was slightly reduced^15^. The elevated [CO_2_] induced changes in grain mineral concentration have been well studied, and lowered nutritional value of wheat by raising atmospheric [CO_2_] is significant^1^,^18^,^19^,^20^. Yet, to date the combined effects of elevated [CO_2_] and soil warming on wheat grain mineral status have not been well illustrated.

Therefore, the objective of this experiment was to explore the mechanisms by which elevated [CO_2_] and soil warming, individually and in combination, affect the mineral nutrition in wheat grain. It was hypothesized that: (i) combination of elevated [CO_2_] and soil warming would improve grain yield while reduce grain mineral concentration; (ii) the xylem transport of nutrients would be decreased by this combination due to lowered stomatal conductance and decreased mineral concentration in the xylem sap, both are responsible for the reduced mineral concentration in wheat grains.

## Results

### Grain yield and phenological dates

Grain yield per pot (*P*_*C*_ = 0.003) and kernel per spike (*P*_*C*_ < 0.001) were significantly increased by elevated [CO_2_] (Table 1). Soil temperature had no significant effects on grain yield, kernel number per spike, spike number per pot and thousand-kernel weight, neither did the interaction between [CO_2_] and soil temperature treatments. In addition, the dates of anthesis and maturity were not significantly affected by elevated [CO_2_] and soil warming (Data not shown).

### Mineral concentrations in wheat organs

**Figure 1:**
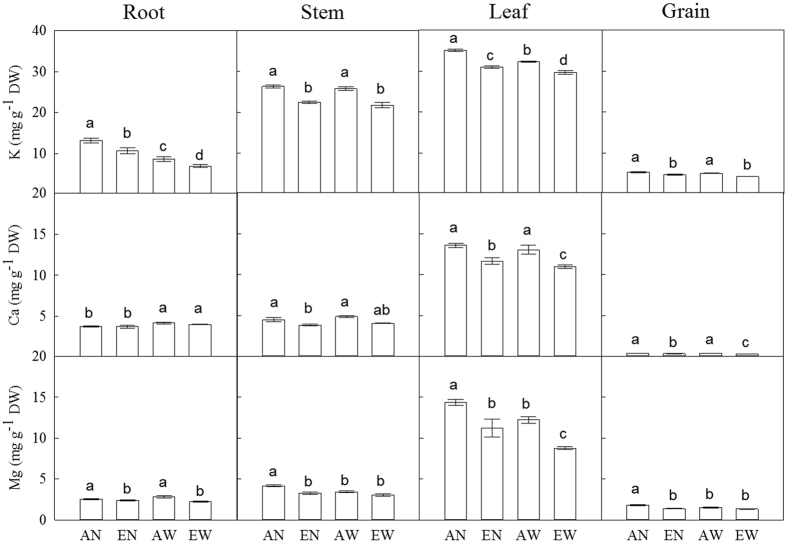
Effects of CO_2_ elevation and soil warming on concentrations of K, Ca and Mg among organs in wheat. Mean values ± SE for each combination of CO_2_ level (A = ambient, E = elevated) and soil temperature (N = non-warmed, W = warmed) are shown (n = 4). Different small letters indicate significant difference at *P *< 0.05 level.

The concentrations of K, Ca and Mg in leaves were the highest, while those in the grains were the lowest among the organs (Fig. 1). Elevated [CO_2_] and soil warming alone decreased K concentration in leaves and roots, and this effect was much stronger with their combination (*P*_*C*×*T*_ < 0.001). In both soil warmed and unwarmed pots, elevated [CO_2_] decreased K concentration in the stems and grains, while soil warming alone had no significant effect. Higher Ca concentration in roots was found in the soil warming treatments (i.e., AW and EW), but no significant difference was found between the control (AN) and soil warming treatments in stem Ca concentration. Soil warming reduced Ca concentration in leaves and grains under elevated [CO_2_], but had no effect on the leaf and grain Ca concentrations under ambient [CO_2_]. In addition, the combination of elevated [CO_2_] and soil warming strongly decreased Ca concentration in the leaves and grains (*P*_*C*×*T*_ < 0.001). Root Mg concentration was higher in plants grown under ambient [CO_2_] than those grown under elevated [CO_2_]. In the stems and grains, Mg concentration was decreased by either elevated [CO_2_] or the combination of elevated [CO_2_] and soil warming. In addition, leaf Mg concentration was also decreased by elevated [CO_2_] and soil warming treatments.

### Plant K, Ca and Mg accumulation

**Figure 2:**
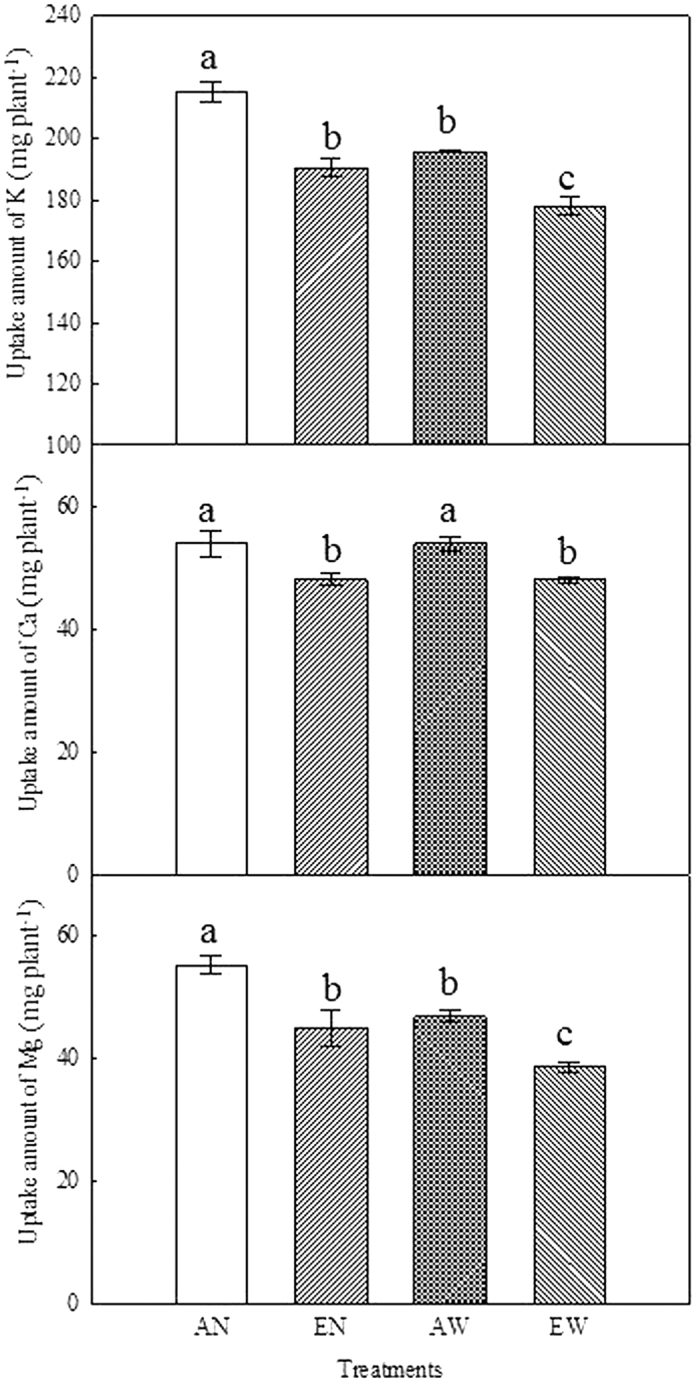
Effects of CO_2_ elevation and soil warming on K, Ca and Mg accumulation in wheat plants. Mean values ± SE for each combination of CO_2_ level (A = ambient, E = elevated) and soil temperature (N = non-warmed, W = warmed) are shown (n = 4). Different small letters indicate significant difference at *P* < 0.05 level.

Compared to the control (AN), K accumulation was decreased by 11.5, 9.0 and 11.3% in EN, AW, and EW plants, respectively (Fig. 2). In relation to the AN plants, Ca accumulation was decreased by 10.7 and 10.9% in EN and EW plants, respectively; but was not affected by soil warming alone (Fig. 2). Plant Mg accumulation was influenced by the treatments in a similar pattern as for K; EN, AW and EW treatments decreased Mg accumulation by 18.5, 15.3 and 30.1%, respectively, as compared to the AN control (Fig. 2).

### Changes in K, Ca, Mg concentrations during grain filling

**Figure 3:**
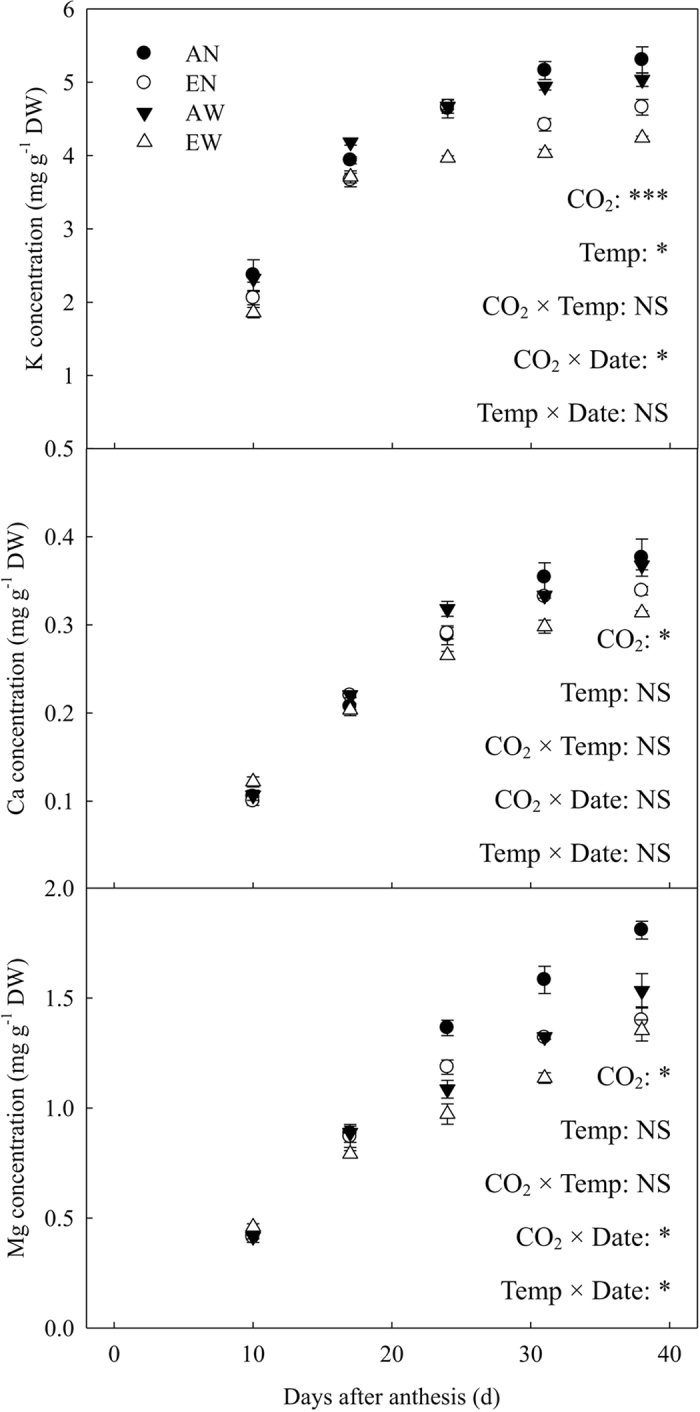
Effects of CO_2_ elevation and soil warming on concentrations of K, Ca and Mg in wheat grain during grain filling. Mean values ± SE for each combination of CO_2_ level (A = ambient, E = elevated) and soil temperature (N = non-warmed, W = warmed) are shown (n = 4). *^,^***indicate significant at *P* < 0.05 and *P* < 0.001, respectively; NS, not significant.

Changes of the concentrations of K, Ca and Mg in the developing grains, measured five times during grain filling as affected by elevated [CO_2_] and soil warming are shown in Fig. 3. Elevated [CO_2_] significantly decreased K concentration in the developing grains on 17 and 31 DAA and the mature grains. The soil warming induced reduction of K concentration was significant from 31 DAA to maturity. However, the interactive effect of elevated [CO_2_] and soil warming was not statistically significant. Elevated [CO_2_] significantly decreased Ca concentration in the developing grains from 23 DAA onwards, whereas the decrease in Ca concentration caused by soil warming was not significant during grain filling. Mg concentration was significantly decreased by the combination of elevated [CO_2_] and soil warming from 17 DAA to maturity, and which was mainly ascribed to the negative effect of elevated [CO_2_] on grain Mg concentration. Compared to the control plants, the reduction in Mg concentration under elevated [CO_2_] become larger in both warmed and unwarmed pots from 17 DAA onwards.

Neither [CO_2_] nor soil temperature had significant effect on grain dry weight resulting in similar dry weight of mature grains among these treatments (Table 1). However, the grain filling rate was seemingly greater in plants grown under elevated [CO_2_] than those grown under ambient [CO_2_], causing an earlier cessation of dry weight accumulation (Data not shown). Grain moisture content declined steadily from 10 DAA onwards and dropped rapidly after 31 DAA (Data not shown). The grain moisture content was approximately 20% at maturity in all treatments. It was interesting that elevated [CO_2_] increased the grain moisture content on 24 and 31 DAA. Whilst, soil warming decreased the grain moisture content significantly on 17 and 31 DAA.

### Stomatal pore aperture and stomatal conductance

**Figure 4:**
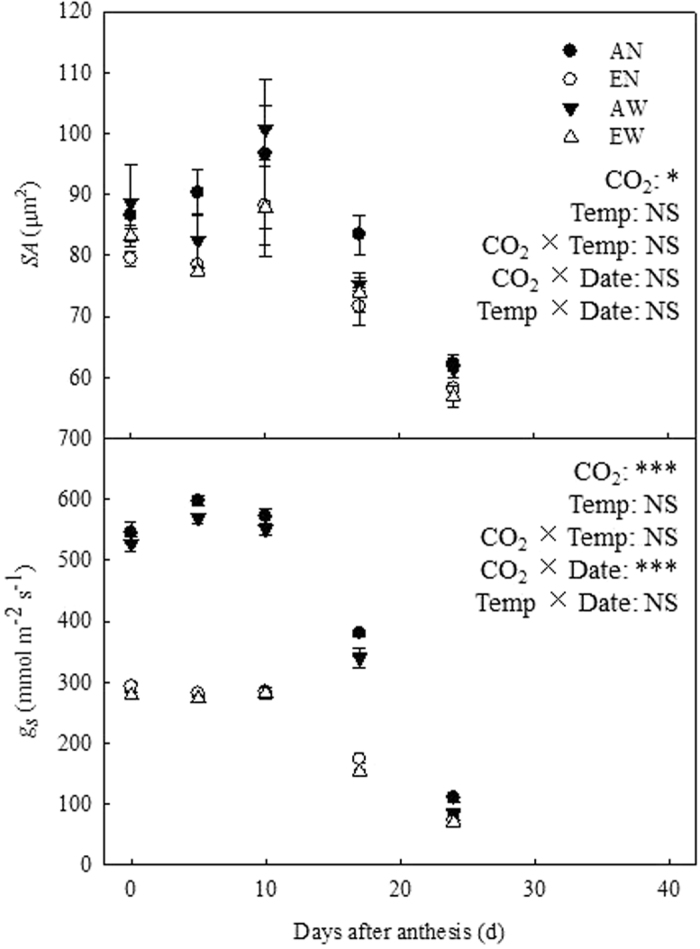
Effects of CO_2_ elevation and soil warming on stomatal pore aperture (*SA*) and stomatal conductance (*g*_*s*_) of wheat leaves during grain filling. Mean values ± SE for each combination of CO_2_ level (A = ambient, E = elevated) and soil temperature (N = non-warmed, W = warmed) are shown (n = 4). *,*** indicate significant at *P* < 0.05 and *P *< 0.001, respectively; NS, not significant.

Figure 4 shows the *SA* remained unchanged till 17 DAA, thereafter it decreased gradually in all treatments. Three-way ANOVA shows that *SA* was only affected significantly by [CO_2_] treatment. The *SA* was the highest in AN, and followed by AW, and the lowest in EN and EW. Under ambient [CO_2_], *g*_*s*_ increased initially, peaking around 5 DAA and tapering off gradually afterward; while for plants grown under elevated [CO_2_] the decrease in *g*_*s*_ occurred after 10 DAA. Elevated [CO_2_] significantly reduced the *g*_*s*_ of flag leaves, while soil warming had no significant effect on the *g*_*s*_: the *g*_*s*_ of AN plants was the highest followed by AW, and the lowest in EN and EW.

### Correlations between stomatal conductance and stomatal pore aperture

**Figure 5:**
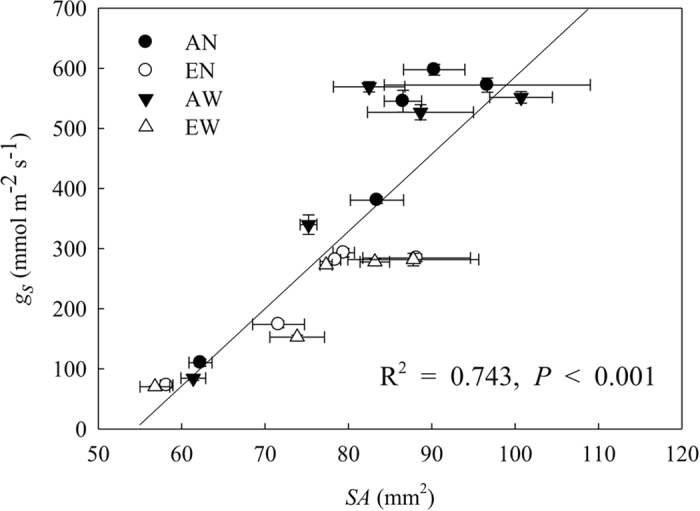
Correlations between stomatal pore aperture (*SA*) and stomatal conductance (*g*_*s*_) under elevated CO_2_ and soil warming treatments. Mean values ± SE for each combination of CO_2_ level (A = ambient, E = elevated) and soil temperature (N = non-warmed, W = warmed) are shown (n = 4).

Significant positive linear relationship between *g*_*s*_ and *SA* was found across the four treatments during grain filling (Fig. 5).

### Ionic concentrations in the xylem sap

The concentrations of K^+^, Ca^2+^ and Mg^2+^ in the xylem sap of wheat plants were all significantly decreased by elevated [CO_2_] and soil warming (Table 2). In relation to the AN controls, the reductions of K^+^, Ca^2+^ and Mg^2+^ concentrations in the xylem sap were 6.5, 12.6 and 20.6%, respectively, in the EN plants. The lowest concentration of these ions was found in plant grown under the combination of elevated [CO_2_] and soil warming. Compared to the AN treatment, EW treatment decreased the concentrations of K^+^, Ca^2+^ and Mg^2+^ by 28.2%, 28.6% and 32.2%, respectively.

## Discussion

### Effects of [CO_2_] elevation on wheat

Compared to the AN plants, the grain yield increased by 6% in plants grown under elevated [CO_2_], which was mainly due to an increase in kernel number per spike. Improved photosynthate availability under elevated [CO_2_] resulting in an increased source activity could have contributed to the greater kernel number in plants grown under elevated [CO_2_]^21^. It has been demonstrated that the potential for increase of grain yield in cereals grown at elevated [CO_2_] is temperature dependent^9^. Recent studies have shown that grain yield of wheat increases in a climate scenario that combines elevated [CO_2_] (700 μmol l^−1^, Tunnel houses) and increased air temperatures (+2 °C, 4 °C or 6 °C) moderately above the current ambient temperature^22^.

In the present study, elevated [CO_2_] did not result in greater grain size, in line with previous findings in wheat^21^,^22^,^23^,^24^. However, it was noticed that the grain filling rate was slightly greater in plants grown under elevated [CO_2_], causing an earlier cessation of grain weight accumulation (Data not shown). It is well known that the grain moisture content is closely related to the grain filling process and thus the final grain weight^25^,^26^. Here, the grain moisture content was higher in the plants grown under elevated [CO_2_] than those grown under ambient [CO_2_] on 24 and 31 DAA, which could be due to a greater osmotic potential gradient for water deposition into to the grain^25^ caused by higher concentration of sucrose under elevated [CO_2_]. Also, higher grain moisture content may indicate a greater sink capacity (i.e., more endosperm cells) of the grains^26^ and might result in a bigger grain size at harvest. However, this was apparently not the case. On the contrary, it was found that soil warming treatment decreased grain moisture content on 17 and 31 DAA, which may be associated with a relatively smaller sink of the grains and thus a lower grain size. Again, this was not true as the final grain weight was identical among all treatments. The reasons behind these inconsistencies remain unknown.

It has frequently been observed that [CO_2_] elevation reduces mineral concentration in plant organs, while increasing the total mineral uptake due to greater plant biomass accumulation^26^. This has led to the notion that the lowered mineral concentration in plant grown under elevated [CO_2_] was ascribed to the dilution by the enhanced plant growth^5^. In the present study, both the concentrations of K, Ca, and Mg in the wheat organs and the total accumulations of K, Ca, and Mg in the plants were significantly decreased by [CO_2_] elevation, indicating that the reduced mineral concentrations was not due to dilution by an enhanced growth. This was particularly the case for the grain, as [CO_2_] elevation had not influenced on the final grain weight, but the K, Ca and Mg concentrations of the grain were significantly lowered. Thus, it is apparent that dilution by greater grain biomass is not the primary reason for reduction in grain mineral concentration under elevated [CO_2_]. In addition, in the developing grains, the concentrations of K, Ca, and Mg in plant grown under elevated [CO_2_] become lower than those of the AN plants since 10, 23 and 23 DAA, respectively, indicating the translocation of K to the grain during grain filling was more sensitive to [CO_2_] elevation.

Plant mineral nutrition is determined by both nutrient acquisition by roots and nutrient transport and partitioning among plant organs^8^. In the present study, the accumulations of K, Ca and Mg in wheat were decreased by elevated [CO_2_], and the reductions were more profound for Mg, followed by K and Ca the least. It is widely recognized that the elevated [CO_2_]-induced decrease in plant mineral uptake might be related to the decreased mass flow or diffusion of mineral ions from the soil solution to the root surface due to lower transpiration rate and/or the reduced ability of mineral acquisition by the roots^6^,^26^. In literature, there is evidence, although variable, that elevated [CO_2_] enhances the root growth and root length density, and which should enable the plants to acquire more nutrients^11^,^27^,^28^. A positive effect of elevated [CO_2_] on root growth was also noticed in the present study (Data not shown), which however did not result in higher mineral uptake in the plants as exemplified by the lower mineral concentration in the xylem sap and the reduced total mineral accumulation in the plants. Therefore, lowered transpiration rate and/or root nutrient acquisition ability could have been the main reasons for the decreased mineral uptake in wheat plants grown under elevated [CO_2_]. In the present study, although the plant transpiration rate was not determined, the lowered g_s_ due to stomatal closure in the wheat plants grown under elevated [CO_2_] was evident. It is well known that elevated [CO_2_] reduces *SA* via depolarizing the guard cell membrane potential, hence leading to stomatal closure^29^,^30^. In this study, elevated [CO_2_] significantly decreased the *SA* and had no significant effect on stomatal density (data not shown) during grain filling in wheat. It has been reported that the short-term changes in *SA* likely determines most of the long-term response of *g*_*s*_ to elevated [CO_2_]^31^. Also, it was found that along with the progress of senescence of the flag leaf, both g_s_ and *SA* were declined and the differences between the treatments were diminished on 24 DAA. Accordingly, elevated [CO_2_] induced partial stomatal closure might have reduced the transpiration rate of the wheat plants resulting in decreased mass flow of the minerals to the root surface^6^, which in turn reduced the root nutrient uptake leading to significant lower concentrations of K^+^, Ca^2+^ and Mg^2+^ in the xylem sap. In good agreement with this, a recent study on wheat reported that the decrease of uptake of Ca and Mg under [CO_2_] elevation is not only by a reduced transpiration flow, but is further reduced by lowered Ca^2+^ and Mg^2+^ concentrations in the xylem^6^. However, it has been proposed that the effect of transpiration rate on mineral uptake is more pronounced with those primarily transported via mass flow (e.g., Ca and Mg) than those via diffusion (e.g., P and K)^5^. Consistent with this, here the reduction of xylem sap K^+^ concentration (6.4%) caused by elevated [CO_2_] was much less compared to those for Ca^2+^ (12.6%) and Mg^2+^ (20.6%). The lowered transpiration rates (as exemplified by the reduced g_s_) and in combination with the decreased mineral concentrations in the transpiration flux (i.e., the xylem sap) could have caused reductions in mineral accumulation in the wheat plants in response to [CO_2_] elevation. In addition, here the effect of [CO_2_] elevation on plant Ca (10.6% reduction) accumulation was found to be less profound than that on Mg (18.6% reduction), which coincided with the reductions in the concentrations of those ions in the xylem sap. However, the 6.4% reduction of K^+^ concentration in the xylem could not fully explain the 10.7% reduction of K accumulation in the plant caused by elevated [CO_2_]. Thus, other factors relating to K metabolisms in the plant might have been involved. We proposed that the relative small reduction of K concentration in the xylem sap of wheat could be a result of shoot-derived K recycled in the phloem and reloaded into the xylem in the roots^32^.

### Effects of soil warming on wheat

In the present study, a slight reduction in grain yield was found with soil warming, where the difference was statistically insignificant. Consistent with this, Weldearegay *et al.*^23^ also found that soil warming only decreased seed set but had no significant effect on grain yield in spring wheat cultivars. Soil warming effect on mineral uptake could be attributed to its effects on root and shoot growth^17^, on the bioavailability of soil nutrients under higher soil temperature^15^, or on the root nutrients uptake capacity^33^. Studies have shown that an increase in the root temperature results in an increase in K concentration but a decrease in Ca concentration of the xylem sap^34^. Compared with Ca and Mg, uptake of K is often more affected by root zone temperatures^35^. Here, an increase of soil temperature by 2.4 °C significantly decreased K^+^, Ca^2+^, and Mg^2+^ concentrations in the xylem sap, and the reduction was greater for Mg^2+^ (22.9%) than for K and Ca (both ca. 15%), contrasting to earlier findings^34^. It has been reported that the optimal temperature for root growth of wheat is lower than 20 °C^36^. In the present study, the day/night temperature in the greenhouse cells were 25/16 ± 2 °C, if the soil temperature was the same, then 2.4 °C increase of soil temperature in the AW treatment would result in soil temperature much higher than the optimal, i.e., 20 °C, thus might have negatively affected the root growth of the wheat plants causing a reduction of acquisition area for mineral nutrients. This may partially account for the reduced mineral concentration in the xylem sap. Besides, the total K, Ca, and Mg accumulated in the AW plants was 9.0, −0.2, and 15.3%, respectively, lower than those of the AN plants, indicating that plant Ca accumulation was unaffected by soil warming alone. However, the reasons for the different effect of elevated [CO_2_] on wheat K, Ca, and Mg accumulation remain unknown. In the developing grains, in relation to the AN treatment, only Mg concentration was significantly lowered by soil warming since 24 DAA; while the concentrations of K and Ca were identical between the two treatments.

### Combined effect of [CO_2_] elevation and soil warming on wheat

Results of this study have clearly shown that the mineral concentrations in the organs (except for Ca in root) and in the xylem sap, and the total mineral accumulation of wheat plants were all significantly reduced by the combined [CO_2_] elevation and soil warming treatment. For most of the variables observed, the combined effects were more pronounced than the individual effect of [CO_2_] elevation or soil warming, especially for the mineral concentrations in the leaf, in the xylem sap, and the total K and Mg accumulation in the plants, where soil warming had exacerbated the negative effect of elevated [CO_2_] on those variables. Although neither of the grain yields components was affected by the combination of [CO_2_] elevation and soil warming, the dramatic effect on mineral concentrations of the wheat plants particularly those in the grains will reduce the grain quality in terms of human nutrition. Thus, selection of wheat cultivars with greater mineral uptake efficiency will of strategic importance to sustain wheat production and grain quality in future climate change scenarios.

## Conclusion

The elevated [CO_2_] increased wheat grain yield due to higher grain number per spike, but reduced the concentrations of K, Ca and Mg in the grain and plant organs. The decreased grain mineral concentration was caused primarily by decreased mineral uptake and was not due to a dilution effect. Elevated [CO_2_]-driven stomatal closure resulted in lower *g*_*s*_, exerted an important role in reduction of mineral uptake, especially for Ca and Mg, in wheat plant. On the other hand, the decreased mineral concentration in xylem sap which could be partially resulted from a reduced capacity of root nutrients uptake might also be responsible for the reduced mineral nutrition in wheat plants. These results documented that future higher [CO_2_] and warmer soil conditions would decrease the dietary availability of K, Ca and Mg from wheat crops.

## Methods

### Experimental setup

A pot experiment was conducted from February to July 2014 in CO_2_ controlled greenhouses (Agrotech, Taastrup, Denmark) in Faculty of Science, University of Copenhagen, Taastrup, Denmark. Eight selected seeds of winter wheat (cv. Lianmai6) were sown per pot (25 cm in height and 15.2 cm in diameter) filled with 2.4-kg peat material (Sphagnum, 32% organic matter, pH = 5.6–6.4 and EC = 0.45 ms cm^−1^). After sowing, half of the pots in each greenhouse were placed on a heating carpet as a sub-group and the soil temperature was increased by 2.4 °C compared with another sub-group^22^. Four seedlings were retained after thinning at the 3^rd^ leaf stage. The pots in the same sub-group were rotated on a daily basis to avoid pseudo replication and heterogeneity of environment inside the greenhouse. The pots were watered daily to keep the soil at an optimal moisture condition (i.e., 80–85% soil relative water content). At jointing stage, all plants were drip irrigated with nutrient solution (in total 2 g N, 1 g P and 1.4 g K were applied to each pot) to prevent any deficiency of nutrients. The photosynthetic active radiation (15 h photoperiod and >500 μmol m^−2^ s^−1^) was supplied by sunlight plus metal-halide lamps. The air temperature and relative humidity were 25/16 ± 2 °C (day/night) and 60 ± 5% in the cells, respectively.

### Treatments

Each sub-group served as a combination of atmospheric [CO_2_] and soil temperature regime, the four treatments are: (i) Ambient [CO_2_] (380 μmol l^−1^) + normal soil temperature (AN); (ii) Elevated [CO_2_] (700 μmol l^−1^) + normal soil temperature (EN); (iii) Ambient [CO_2_] (380 μmol l^−1^) + soil warming (+2.4 °C) (AW); (iv) Elevated [CO_2_] (700 μmol l^−1^) + soil warming (+2.4 °C) (EW).

### Sampling and mineral concentration analysis

On 10, 17, 24 and 31 days after anthesis (DAA), wheat grains were harvested and weighed (g fresh weight, g FW), and then dried at 70 °C in an oven to constant weight for determining the dry weight (g dry weight, g DW). Grain moisture content was calculated according to grain fresh weight and dry weight. At each sampling date, four independent samples, each composed of four plants, were collected. At maturity, the plants were separated into root, stem, leaf and grain for mineral analysis. Concentrations of K, Ca and Mg were analyzed after high-pressure digestion with nitric acid (UltraClave III, MLS, Leutkirch, Germany) using inductively-coupled plasma optical emission spectrometry (ICP-OES 720, Varian, Palo Alto, CA, USA). At maturity, grain was harvested, and spike number, kernel per spike and thousand-kernel weight were determined on 4 pots from each treatment.

### Xylem sap collection and ionic concentration analysis

For xylem sap collection, the entire pot was sealed into a Scholander-type pressure chamber and all stems were faggoted and detopped at 10 cm from the stem base. Xylem sap was collected on 10 DAA using a pipette from the cutting surface into an Eppendorf vial wrapped with aluminium foil. All sap samples were frozen immediately in liquid nitrogen after sampling and stored at −80 °C until analysis. Concentrations of K^+^, Ca^2+^ and Mg^2+^ in the xylem sap were determined by ion chromatography (Metrohm AG, Herisau, Switzerland) using a Metrosep C4-100 analytical column (4 × 125 mm, 1.7 mM nitric acid/0.7 mM dipicolinic acid eluent)^32^.

### Stomatal conductance

Stomatal conductance (*g*_*s*_) of flag leaves was measured using a leaf porometer (Decegon Devices, Pullman, WA, USA) at anthesis and on 5, 10, 17 and 24 DAA.

### Stomatal pore aperture

Epidermal impressions of both adaxial and abaxial leaf surfaces were taken from three flag leaves in each treatment at anthesis and on 5, 10, 17 and 24 DAA. Fingernail polish imprints were obtained halfway from the leaf tip to the base of each leaf, and using clear cellophane tape to transfer the ‘impression’ to a microscope slide^37^. The imprints were observed under a LEITZ DMRD microscope camera system (Leica Microscope and System GmbH, D35530, Wetzlar, Germany) equipped with a digital camera, and the images were presented using image-editing software (Leica Microsystems, version 2.5.0, CMS GmbH, Switzerland) on a computer screen. Stomatal pore aperture length (*L*_*a*_) and stomatal pore aperture width (*W*_*a*_) were measured with the images using UTHSCSA ImageTool software (UTHSCSA Image Tool for Windows version 3.00). Stomatal pore aperture (*SA*) was calculated according to Doheny-Adams *et al.*^38^ as the following equations:


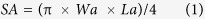


where *W*_*a*_ is pore aperture width, *L*_*a*_ is pore aperture length.

### Statistical analysis

For *SA* and concentrations of K^+^, Ca^2+^ and Mg^2+^ in the xylem sap and in different organs and plant total K, Ca, and Mg accumulation amount, the interactive effect between [CO_2_] and soil temperature was analyzed by two-way ANOVA using the Duncan test (*P* < 0.05) (SigmaSATA, V3.5, Systat Software, CA, USA). For grain dry weight, moisture content, *g*_*s*_ and grain concentrations of K, Ca^2+^ and Mg^2+^ during grain filling, post hoc ANOVAs were used to illustrate significant CO_2_ × soil temperature, CO_2_ × date or soil temperature × date interactions (SPSS version 20.0 for windows, IBM SPSS Statistics, Chicago, IL, USA).

## Additional Information

**How to cite this article**: Li, X. *et al.* Soil warming enhances the hidden shift of elemental stoichiometry by elevated CO_2_ in wheat. *Sci. Rep.* **6**, 23313; doi: 10.1038/srep23313 (2016).

## Figures and Tables

**Table 1:** Grain yield per pot, spike number per pot, kernel number per spike, and thousand kernel weight (TKW) for each combination of CO_2_ level (A = ambient, E = elevated) and soil temperature (N = non-warmed, W = warmed).

Yield issue	Treatments				Analysis of variance		
	AN	EN	AW	EW	CO_2_	Temp	CO_2_ × Temp
Grain yield (g pot^−1^)	20.4 ± 0.4	21.6 ± 0.4	20.2 ± 0.1	21.3 ± 0.3	*	NS	NS
Spikes pot^−1^	15.8 ± 0.5	16.0 ± 0.7	16.2 ± 0.5	16.0 ± 0.4	NS	NS	NS
Kernels spike^−1^	35.3 ± 0.3	37.1 ± 0.4	34.8 ± 0.3	36.9 ± 0.5	***	NS	NS
TKW (g)	35.0 ± 0.1	34.9 ± 0.2	34.5 ± 0.2	35.1 ± 0.1	NS	NS	NS

Mean values ± SE for each combination of CO_2_ level and soil temperature are shown (n = 4). *^,^***indicate significant at *P* < 0.05 and *P* < 0.001, respectively; NS, not significant.

**Table 2:** Output of two-way ANOVA for concentrations of K^+^, Ca^2+^ and Mg^2+^ in the xylem sap of wheat plant for each combination of CO_2_ level (A = ambient, E = elevated) and soil temperature (N = non-warmed, W = warmed) at 10 days after anthesis.

Mineral (mg L^−1^)	Treatments				Analysis of variance		
	AN	EN	AW	EW	CO_2_	Temp	CO_2_ × Temp
K^+^	732.6 ± 12	685.3 ± 9	621.1 ± 35	525.9 ± 18	***	***	NS
Ca^2+^	167.8 ± 3	146.7 ± 3	129.4 ± 11	119.8 ± 5	*	***	NS
Mg^2+^	112.9 ± 6	89.6 ± 4	80.4 ± 9	76.6 ± 4	*	*	NS

Mean values ± SE for each combination of CO_2_ level and soil temperature are shown (n = 4). *^,^***indicate significant at *P* < 0.05 and *P* < 0.001, respectively; NS, not significant.
