# Corrigendum: Soil warming enhances the hidden shift of elemental stoichiometry by elevated CO_2_ in wheat

**DOI:** 10.1038/srep46921

**Published:** 2017-12-22

**Authors:** Xiangnan Li, Dong Jiang, Fulai Liu

Scientific Reports 6: Article number: 23313; 10.1038/srep23313 published online: 03 22 2016; updated: 12 22 2017.

The authors neglected to cite two previous studies related to the effect of elevated CO_2_ on minerals. These additional references are listed below as references 1 and 2, and should appear in the text as below.

In the Introduction section,

“However, the human nutrition depending on wheat grains might be threatened by the climate change.”

should read:

“However, the human nutrition depending on wheat grains might be threatened by the climate change^1^,^2^.”
